# Mitochondrial Lactate Dehydrogenase Is Involved in Oxidative-Energy Metabolism in Human Astrocytoma Cells (CCF-STTG1)

**DOI:** 10.1371/journal.pone.0001550

**Published:** 2008-02-06

**Authors:** Joseph Lemire, Ryan J. Mailloux, Vasu D. Appanna

**Affiliations:** Department of Chemistry and Biochemistry, Laurentian University, Sudbury, Ontario, Canada; University of California, Berkeley, United States of America

## Abstract

Lactate has long been regarded as an end product of anaerobic energy production and its fate in cerebral metabolism has not been precisely delineated. In this report, we demonstrate, for the first time, the ability of a human astrocytic cell line (CCF-STTG1) to consume lactate and to generate ATP via oxidative phosphorylation. ^13^C-NMR and HPLC analyses aided in the identification of tricarboxylic acid (TCA) cyle metabolites and ATP in the astrocytic mitochondria incubated with lactate. Oxamate, an inhibitor of lactate dehydrogenase (LDH), abolished mitochondrial lactate consumption. Electrophoretic and fluorescence microscopic analyses helped localize LDH in the mitochondria. Taken together, this study implicates lactate as an important contributor to ATP metabolism in the brain, a finding that may significantly change our notion of how this important organ manipulates its energy budget.

## Introduction

Since lactate is produced during anaerobic energy metabolism, this monocarboxylic acid has been regarded as a by-product of this process. Its accumulation has been linked to a variety of abnormalities such as plasma acidosis and muscle fatigue. However, recent studies seem to implicate this monocarboxylic acid in aerobic energy production [1]–[3]. The ability of skeletal muscle to consume lactate oxidatively, and the presence of LDH in the mitochondria have been invoked to validate the involvement of lactate in the tricarboxylic acid (TCA) cycle and the oxidative energy-generating machinery [4]–[6]. The occurrence of a lactate shuttle designed to promote mitochondrial energy metabolism has been subsequently proposed [4], [7]. The presence of a monocarboxylate transporter 1 (MCT 1) and the participation of lactate-derived pyruvate in the mitochondria appear to support the notion that lactate may be contributing to oxidative energy formation in muscles [8]. The involvement of a lactate shuttle in peroxisomes confers this organelle with the ability to metabolize fatty acids. The presence of this enzyme was recently confirmed in liver peroxisomes [9], [10]. The possible existence of mitochondrial LDH in mammalian spermatozoa has been suggested and its participation in the production of oxidative energy has been reported [11], [12].

Although, the brain constitutes 2–3% of the body's mass, it does consume high amounts of glucose, mostly in the production of ATP for the maintenance of the cerebral ion gradient [13]. As the brain is known to produce lactate under aerobic conditions, it has been postulated that this monocarboxylic acid may be supporting oxidative respiration in the neurons. This may be critical in the functioning of the brain as this organ stores little fuel and depends on a steady supply of metabolic substrates to fulfill its high-energy demanding functions. Astrocytes appear to be the main generator of lactate in the brain, which is apparently channelled to the energy-intensive neurons for oxidative ATP production [14]–[16]. This astrocyte-neuron lactate shuttle has been hypothesized to be critical for the effective functioning of the neurons.

Since neurons are the major consumers of cerebral energy, very little attention has been given to the energy need of astrocytes, cells that outnumber neurons 10∶1, depending on the region of the brain [17], [18]. In this study we have evaluated the ability of an astrocytic cell line to generate oxidative energy from lactate, a substrate that can be astrocytically-derived and is also present normally in the plasma in concentration as high as 1 mM [19]–[21] Here, we report on the presence of a mitochondrial LDH that enables this model astrocyte to utilize lactate in the production of ATP via oxidative phosphorylation. This finding has important implications in the cerebral energy budget and metabolism. It also paves the way to reassess the significance of lactate in brain biochemistry.

## Materials and Methods

### Cell Culture, Isolation, and Fractionation

The human astrocytoma cell line (CCF-STTG1) was acquired from the ATCC, Manassas,Virginia, USA. CFF-STTG1 cells are an adherent cell line established from a grade IV astrocytoma. 70–80% of cells in culture are found to be positive for glial fibrillary acidic protein. This cell line was chosen due to its similarity with normal astrocytes [22]. The astrocytic cell line was sustained in α-Minimum Eagle Media (MEM) supplemented with 5% Fetal Bovine Serum (FBS) and 1% antibiotics (streptomycin, and penicillin). CCF-STTG1 cells were seeded at 1.0×10^5^ cells/mL in 175 cm^2^ culture flasks (2×10^6^ cells/flask), maintained in an incubator with 5% CO_2_ in a humidified atmosphere operating at 37°C. When a confluency of 75% was attained the cell monolayer was rinsed with Phosphate Buffered Saline [PBS; 136 mM sodium chloride, 2.5mM potassium chloride, 1.83mM dibasic sodium phosphate, and 0.43 mM monobasic potassium phosphate (pH 7.4)] and the cultures were re-incubated with serum-free media reconstituted with 2.5 mM lactate, 2.5 mM glucose, or 2.5 mM citrate. The viability of the cells was assessed using the Trypan Blue Exclusion assay [23]. Following a 24 hr incubation period, the media was removed and the cells were washed with PBS. These astrocytes were then harvested by trypsinization and centrifugation at 250 g for 10 min at 4°C. The pelleted cells were resuspended in cell storage buffer (CSB; 50 mM Tris-HCl, 1 mM phenylmethylsulphonylfluoride, 1 mM dithiothreitol, 250 mM sucrose, 2 mM citrate, containing 0.1 mg/mL of pepstatin A and 0.1 mg/mL of leupeptin) and stored at −86°C until needed. When the cells were required, they were thawed and pelleted by centrifugation at 250 g for 10 min at 4°C. The pellet was resuspended in a minimal volume of CSB (50 uL/4.0×10^6^ cells). The resulting cell suspension was disrupted utilizing a Brunswick sonicator, operating for 7 s with 1s bursts. Intact whole cells and nuclei were removed at 850 g for 10 min at 4°C. The mitochondria were isolated from the cytoplasmic portion by centrifugation at 12,000 g for 30 min at 4°C. After the centrifugation, the soluble fraction was then placed in an ice cold microcentrifuge tube and the mitochondrial pellet was resuspended in a minimal amount of CSB. Purity of mitochondria and cytoplasmic portions were determined by immunoblotting for Voltage Dependent Anion Channel (Abcam), for the mitochondria and F-actin (Santa Cruz) for the cytosol. Subfractionation of the mitochondria involved treatment with 1% digitonin for 30 min on ice, proceeded by high speed centrifugation at 10,000 g for 30 min, respectively [24]. The pellet consisted of the inner mitochondrial membrane and the matrix while the supernatant contained the outer membrane and the intermembrane space. The Bradford assay was utilized to quantitate the protein content and Bovine Serum Albumin (BSA) was utilized as the standard [25]. The homogeneity of the sub-fractions of the mitochondria were confirmed utilizing cytochrome C antibodies (Abcam) for the outermembrane/inner membrane space portion, while succinate dehydrogenase antibodies (a gift from Dr. Lemire, University of Alberta) were used for the innermembrane/matrix portion of the mitochondria.

### Metabolite Analysis

To ascertain the consumption of lactate, spent fluid from CCF-STTG1 cell cultures was collected at varying time intervals and analyzed utilizing a Rezex organic acid column (Phenomenex) in an Alliance HPLC (Waters). The mobile phase utilized was 2.5mM H_2_SO_4_ operating at an elution rate of 0.6 mL/min at ambient temperature. Measuring lactate consumption via the mitochondria involved obtaining mitochondrial isolate (2 mg/mL protein equivalent) from the astrocytic cell line and incubating in a phosphate buffer [10 mM phosphate, 5mM MgCl_2_ (pH 7.4)] containing 5 mM lactate or 5 mM citrate, 0.1 mM NAD^+^ for varying time intervals at 37°C. Mitochondria were also incubated with 5 mM lactate, 0.1 mM NAD^+^, in addition to 10 mM oxamate to inhibit LDH [4], [26]. The reaction was stopped via boiling of the samples for 10 min. The organic acids and adenosine nucleotides were subsequently extracted for HPLC analysis. The resultant suspension from the reaction was analyzed using a C_18_-reverse phase column (Phenomenex) with the aid of an Alliance HPLC (Waters). The mobile phase utilized consisted of 20 mM KH_2_PO_4_ (pH 2.9 with 6N HCl), operating at an elution rate of 0.7 mL/min at ambient temperature. To analyze nicotinamide nucleotide levels, the mobile phase was altered to a 20 mM KH_2_PO_4 _(pH 7.0 with 6N HCl) containing 5% acetonitrile to accurately measure NAD(H) levels [27]. The identities of the metabolites were compared with known standards, and the reaction mixtures were spiked with the appropriate standard metabolites. The initial levels of metabolites were obtained by running reaction mixtures at time zero. For confirmation of lactate consumption by the mitochondria, mitochodria isolated from the CCF-STTG1 cells were incubated in phosphate buffer including 10 mM [3-^13^C] lactate (Cambridge Isotope Laboratories, Inc), and 0.1 mM NAD^+^. To monitor TCA cycle intermediate accumulation, 1 µM of NaN_3_ was added to this reaction mixture. Aliquots were collected at varying time intervals, boiled and analyzed. To make certain the metabolites being observed were indeed native to the TCA cycle, aliquots were collected during HPLC at the given retention times and lyophilized. Subsequently, enzyme specific assays were performed on the samples to confirm metabolite identity. To confirm citrate, 200 µL of sample and 10 units of aconitase (Sigma) were placed in equilibration buffer [25 mM Tris-HCl, 5 mM MgCl_2 _(pH 7.4)] and the formation of cis-aconitate was measured at 220 nm. For the confirmation of fumarate, 200 µL of sample and 10 units of fumarase (Sigma) were added to equilibration buffer and the disappearance of fumarate was measured at 220 nm. Succinate confirmation was ascertained utilizing 200 µL of sample, 60 µg/mL of membrane fraction collected from *P. fluorescens*, and 2.5 mg/mL of DCIP (dichloroindophenol) in equilibration buffer. The oxidation of DCIP was measured at 500 nm [28]. All reactions were confirmed with standard metabolites obtained from Sigma. NMR analyses were performed using a Varian Gemini 2000 spectrometer operating at 50.38 MHz for ^13^C [29]. Samples were analyzed with a 5mm dual probe (35° pulse, 1-s relaxation delay,8 kilobytes of data, and 2000 scans). Chemical shifts were referenced to standard compounds under analogous conditions.

### Blue Native PAGE (BN PAGE) and Protein Activity Assays

BN PAGE was performed following the method described in [30], [31]. 4–16% gradients gels were cast in a BioRad MiniProtean™ 2 electrophoresis unit. Samples of 2 µg of protein equivalent/µL were prepared in blue native buffer [500 mM 6–amino hexanoic acid, 50 mM BisTris (pH 7.0), and 1% β–dodecyl-D-maltoside]. When soluble proteins were being dealt with β-dodecyl-D-maltoside was omitted. Each well of the native gel was loaded with 30 µg of prepared protein samples. The blue cathode buffer [50 mM Tricine, 15 mM BisTris, 0.02% w/v Coomassie G-250 (pH 7.0) at 4°C] was exchanged for colourless cathode [50 mM Tricine, 15 mM BisTris (pH 7.0) at 4°C] once the running front reached half-way through the resolving gel. Upon completion, the gel was incubated in an equilibrium buffer [25 mM Tris-HCl, 5 mM MgCl_2_ (pH 7.4)] for 15 min. Enzymatic activity of LDH was visualized with the aid of formazan precipitation. The gels were incubated in a reaction mixture consisting of equilibration buffer, 5 mM lactate, 0–0.5 mM NAD^+^, phenazinemethosulphate (PMS), and iodonitrotetrazolium (INT) in an effort to detect LDH activity. 4 mM Silver Nitrate (AgNO_3_) was included in the reaction mixture in order to identify LDH1 [32]. Standards from porcine heart were used as molecular weight standards. The activity of cytochrome C oxidase was assessed using diaminobenzidene as the chromophore. Equilibration buffer containing 10 mg/mL of diaminobenzidine, 10 mg/mL cytochrome C, and 562.5 mg/mL of sucrose was used to visualize the activity of this enzyme [33].

### Oxygen Consumption by the Astrocytic Mitochondria

To further determine whether lactate was contributing to mitochondrial respiration, oxygen consumption was measured utilizing an Orion ® O_2_ electrode. Isolated mitochondria were incubated with 5 mM substrate (lactate, succinate, or pyruvate), 0.5 mM NAD^+^, and 0.5 mM ADP in equilibration buffer. Oxygen consumption was monitored over a 5min time interval. To confirm the mitochondrial utilization of lactate, 5 mM KCN was included in the reaction mixture.

### Immunoblot Analysis

SDS PAGE and 2D SDS PAGE gels were performed in accordance with the modified method described by [34], [35]. The protein samples were solubilized in 62.5 mM Tris-HCl (pH 6.8), 2% SDS, and 2% β-mercaptoethanol at 100°C for 5 min. Following solubilization the protein samples were then loaded into a 10% isocratic gel and electrophoresed using a discontinuous buffer system. For 2D immunoblot analysis, activity bands from native gels were precision excised from the gel and incubated in denaturing buffer (1% β-mercaptoethanol, 5% SDS) for 30 min, and then loaded vertically into a well of an SDS gel. Following electrophoresis, the proteins were transferred electrophoretically to a Hybond™-Polyvinylidene difluoride membrane for immunoblotting. Non-specific binding sites were blocked by treating the membrane with 5% non-fat skim milk dissolved in TTBS [20 mM Tris-HCl, 0.8% NaCl, 1% Tween-20 (pH 7.6)] for 1 h. Polyclonal antibodies for LDH were obtained from Santa Cruz, and monoclonal antibodies for LDH1 from Abcam. The secondary antibodies (Santa Cruz) consisted of horseradish peroxidase-conjugated mouse anti-goat. The detection relied on incubation of the probed membrane for 5 min at room temperature in the presence of Chemiglow reagent (Alpha Innotech). Visualization of the immunoblot was documented via a ChemiDoc XRS system (Biorad Imaging Systems)

### Fluorescence Microscopy

Localization of LDH to the mitochondria was further confirmed using immunofluorescence. Astrocytes (CCF-STTG1 cells) were grown to a minimal density on coverslips and incubated for 24 h in the presence of serum-free α-MEM containing 2.5 mM lactate. The coverslips were washed once with 0.5 mM EDTA and twice with PBS to ensure all residual lactate was removed. In order to visualize the mitochondria, cells were incubated in Rhodamine B (10 µg/mL in α-MEM) for 20 min at 37°C. The cells were then fixed with methanol:acetic acid (3:1). Following fixation the cells were then incubated in Hoechst 33258 (2.5 µg/mL in PBS) for 10 min at ambient temperature. To probe for LDH, coverslips were submerged in Tween-20 Tris Buffered Saline (TTBS) with 5% FBS for 1 h to block non-specific binding sites. The coverslip was then rinsed 3× with Tris Buffered Saline (TBS). These astrocytic cells were then incubated for 2 h in anti-LDH antibody (1/750) in TBS/5% FBS solution. The coverslips were washed thoroughly and incubated in anti-goat conjugated to fluorescein isothiocyanate (FITC). Following exposure to the antibodies, the coverslip was washed and mounted onto microscope slides. The cells were visualized using an inverted deconvolution microscope (Zeiss). The protonated Rhodamine B molecule was detected at λ_excitation_ = 564 nm and λ_emmision_ = 620 nm. Hoechst 33258 was detected at λ_excitation_ = 360 nm and λ_emmision_ = 470 nm. Similarily, the fluorescein isothiocyanate was detected at λ_excitation_ = 495 nm and λ_emmision_ = 520 nm, allowing for the detection of LDH in the mitochondria of this astrocytic cell line.

### Statistical Analysis

All experiments were performed thrice and in duplicate. Where appropriate the student t test was utilized.

## Results

**Figure 1:**
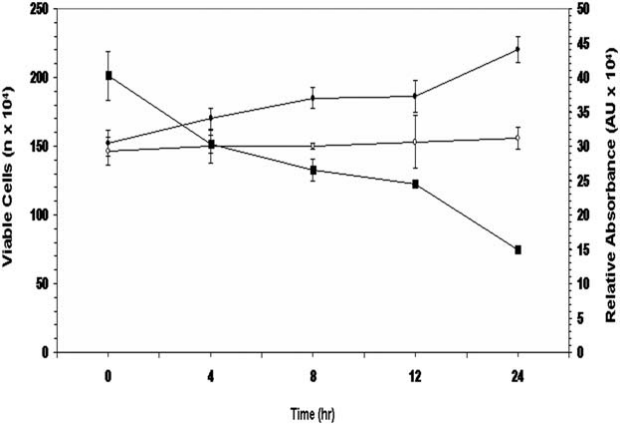
Lactate utilization by an astrocytic cell line. CCF-STTG1 cultures were supplemented with 2.5 mM lactate in serum free media. Lactate measurements were performed using HPLC (Reezex organic acid column) at various time intervals. Viable cell counts were performed using the Trypan Blue Exclusion Assay. • Corresponds to viable cell number of the astrocytic cells grown in serum free α-MEM+2.5 mM lactate. ○ Corresponds to viable cell number of astrocytic cells grown in serum free α-MEM. ▪ Corresponds to relative amount of lactate levels in the spent fluid (lactate cultures). n = 3, mean±SD

**Figure 2:**
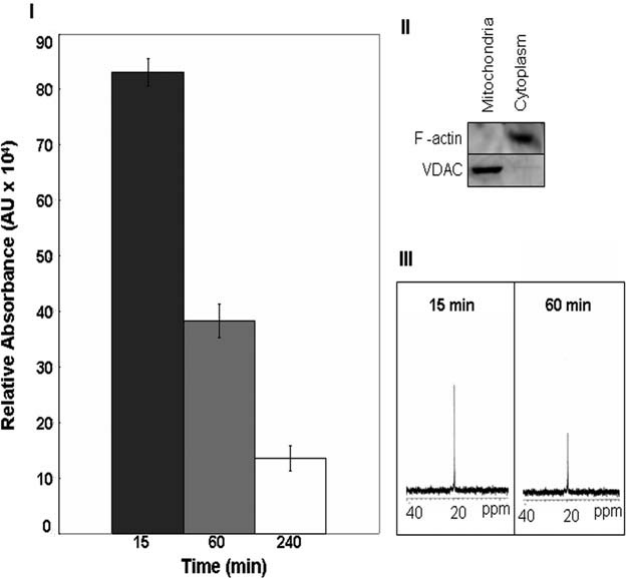
Lactate consumption by mitochondria derived from this astrocytic cell line. I) Mitochondria were incubated with 5mM lactate and 0.1mM NAD^+^. The time dependent consumption of lactate by the mitochondria was measured using HPLC. II) The purity of the mitochondrial and cytoplasmic fractions were confirmed using VDAC and F-actin respectively. III) Mitochondrial lactate consumption was confirmed using NMR spectroscopy. Data were acquired after 15min and 60min respectively. n = 3, mean±SD

As lactate is a cerebral metabolite, it is important to evaluate if this moiety is utilized in an oxidative manner by the brain cells. In this study, when the astrocytic cells (CCF-STTG1) were incubated with lactate, cellular proliferation and consumption of this metabolite were observed. Indeed, the astrocytic cells were able to utilize nearly 25% of the extracellular lactate following a 4 h incubation period. Nearly 75% of the lactate was consumed after 24 h (Figure 1). Skeletal muscle has been shown *a priori* to oxidize exogenous lactate for aerobic energy production [4]. As lactate was readily metabolized, we hypothesized that this process may be mediated in the mitochondria. Purified astrocytic mitochondria were incubated in 5 mM lactate for 240 min at 37°C (Figure 2I). HPLC analysis revealed that up to 85% of the lactate was consumed within 240 min. Reactions with ^13^C-labeled lactate further substantiated the observed metabolism of lactate by the mitochondria. The signal at 19 ppm (corresponding to the label on the C3 position) was diminished following 15 min incubation with the labeled lactate (Figure 2III).

**Figure 3:**
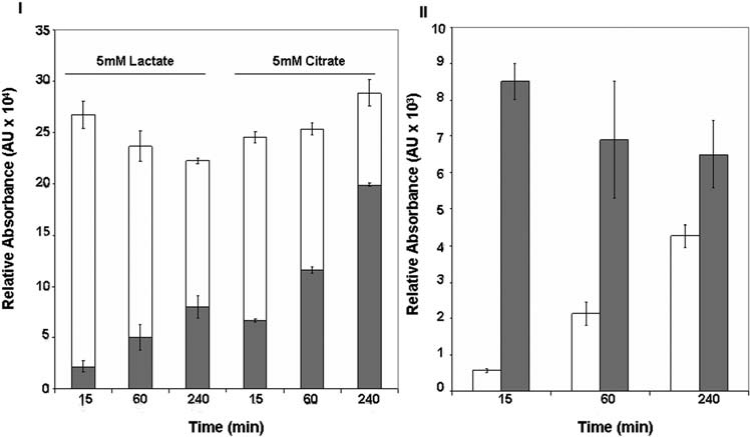
Lactate as a source of mitochondrial energy in an astrocytic cell line. Mitochondria were incubated with 5 mM lactate or 5 mM citrate, 0.1 mM NAD^+^, and 0.1 mM ADP for varying time intervals. Nucleotide levels were measured by HPLC using a C_18_ reverse phase column. I) ATP/ADP ratio in mitochondria. Open bar □ = ADP, and closed bar ▪ = ATP II) NAD^+^/NADH ratio in mitochondria incubated with lactate. Open bar □ = NADH, and closed bar ▪ = NAD^+^. Peaks were confirmed by utilizing known standards and by spiking the samples. n = 3, mean±SD

**Figure 4:**
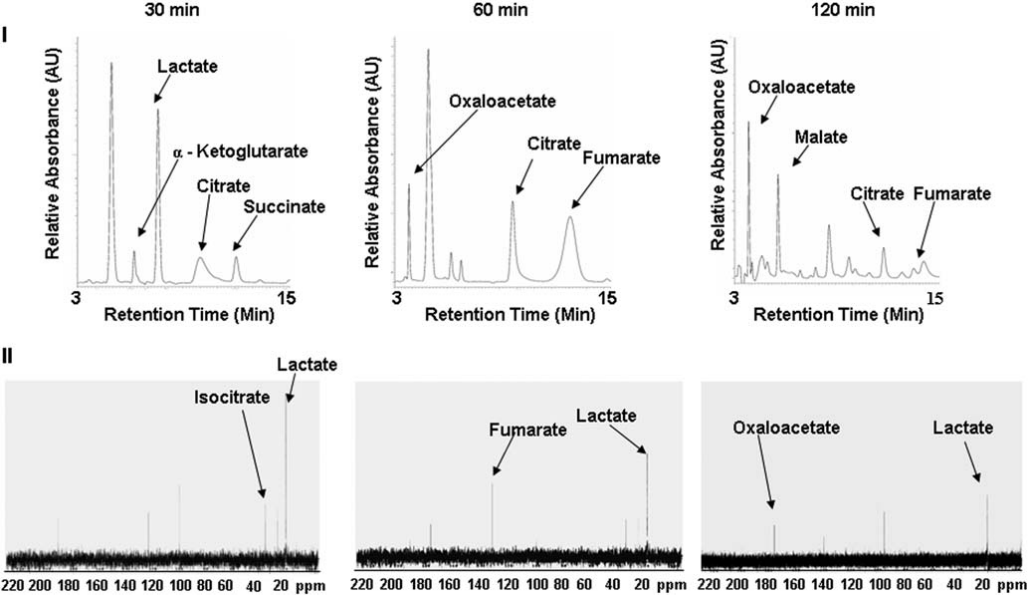
Oxidative metabolism of lactate in astrocytic mitochondria. Mitochondria isolated from CCF-STTG1 cells were incubated in 10 mM ^13^C_3_–lactate, 0.1 mM NAD^+^ and 1 µM NaN_3_ for varying time intervals. Accumulation of TCA cycle intermediates were measured via I) HPLC and II) NMR spectroscopy. HPLC fractions were also confirmed by enzymatic assays (citrate, succinate, and fumarate).

**Figure 5:**
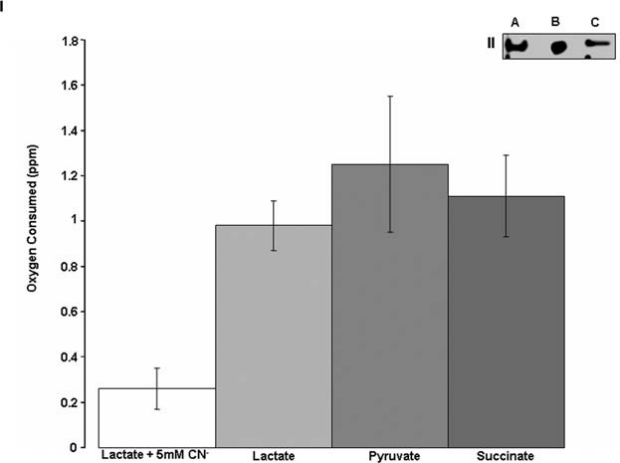
Lactate promotes aerobic respiration in astrocytic mitochondria. I) Mitochondria from an astrocytic cell line were isolated and oxygen consumption was measured over a 5 min period, utilizing an oxygen electrode (Orion®). Mitochondria were incubated with 5 mM substrate, 0.5 mM NAD^+^, and 0.5 mM ADP. A reaction buffer blank was also analyzed to ensure proper instrument calibration. II) In gel activity of cytochrome C oxidase. A) CCF-STTG1 cells incubated with 2.5 mM lactate. B) CCF-STTG1 cells incubated with 2.5mM glucose. C) CCF-STTG1 cells; incubated with 2.5 mM citrate.

Since the mitochondria from these cells were able to metabolize exogenous lactate, it was important to evaluate if this moiety was contributing to the TCA cycle and ATP production. An increase in mitochondrial ATP was recorded with increasing incubation time (Figure 3I). A similar pattern was observed with citrate, a well-known substrate for mitochondrial oxidative phosphorylation. The ability of the mitochondria to generate energy using lactate was evaluated further by assessing the relative levels of NAD^+^ and NADH (Figure 3II). Indeed, an increase in NADH and a concomitant decrease in NAD^+^ was recorded. The ability of the mitochondria to oxidize lactate for the purpose of NADH production implies that this monocarboxylic acid is metabolized via the TCA cycle. Thus, we incubated purified mitochondria in 10 mM ^13^C-labeled lactate and measured the levels of TCA cycle intermediates by HPLC and ^13^C-NMR. HPLC analysis of the mitochondrial reactions revealed that the TCA cycle intermediates oscillated in abundance over the 120 min suggesting that the TCA cycle was fully active in the presence of lactate (Figure 4I). These data were further substantiated with ^13^C-NMR. Indeed, at 30 min a strong lactate signal was apparent whereas fumarate and oxaloacetate became pronounced with incubation time (Figure 4II). As lactate was involved in mitochondrial oxidative metabolism, it became critical to monitor oxygen consumption in mitochondria incubated in lactate. As shown in Figure 5I, these astrocytic mitochondria readily reduced O_2_ to H_2_O in the presence of lactate. The ability of lactate-treated mitochondria to utilize O_2_ was confirmed by incubating these fractions in KCN, a known cytochrome C oxidase inhibitor. Indeed O_2_ consumption was significantly reduced in the presence KCN. In order to confirm that the mitochondria from these astrocytic cells were capable of oxidizing lactate, we measured the activity of cytochrome C oxidase by BN PAGE. The in-gel activity of cytochrome C oxidase from lactate-exposed cells exhibited similar band intensity to cells incubated in 2.5 mM citrate or 2.5 mM D-glucose (Figure 5II). Thus, lactate can be utilized as an aerobic energy source by these astrocytes.

**Figure 6:**
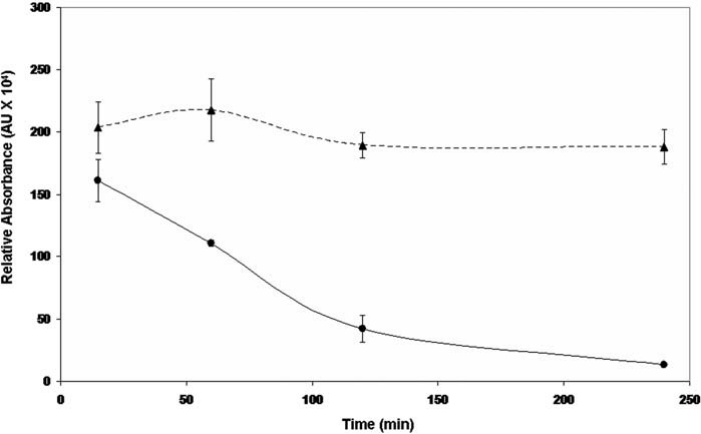
Mitochondrial lactate metabolism. Mitochondria from CCF-STTG1 cells were isolated and incubated with 5 mM lactate, and 0.1 mM NAD^+^ for varying time intervals within the presence or in absence of 10 mM oxamate. Relative lactate levels were measured by HPLC. • = mitochondria in the absence of 10mM oxamate. ▴ = mitochondria in the presence of 10mM oxamate. n = 3; SD

**Figure 7:**
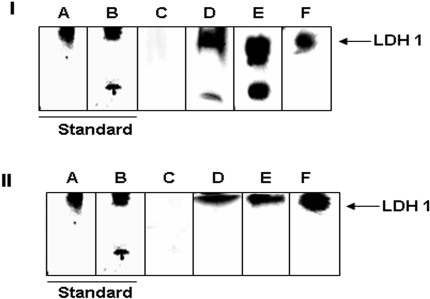
BN PAGE analyses of LDH in an astrocytic cell line. I) Mitochondrial fraction. II) Soluble fraction. A) Coomassie stain for LDH from porcine heart (Sigma). B) Coomassie stain for LDH from porcine muscle (Sigma). C) In gel detection of LDH activity with 0 mM NAD^+^. D) In gel detection of LDH activity with 0.1 mM NAD^+^. E) In gel detection of LDH activity with 0.5 mM NAD^+^. F) In gel detection of LDH activity with 0.5 mM NAD^+^ and 2 mM AgNO_3_.

**Figure 8:**
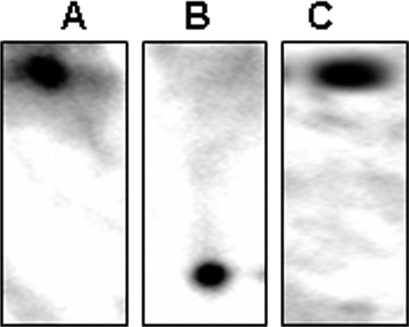
2D immunoblot analysis of LDH. Activity bands were excised from BN PAGE experiment and ran on a 10% SDS-PAGE. A) Upper band (mitochondrial fraction). B) Lower band (mitochondrial fraction). C) Band from soluble fraction

The ability of the mitochondria to utilize lactate as an oxidizable energy source would depend on the presence of LDH within the mitochondria. However, the use of lactate for ATP production in the mitochondria from astrocytes has never been demonstrated. In order to account for the observed increase in ATP production and O_2_ consumption in the presence of lactate, it was important to evaluate whether LDH was critical in this process. This enzyme is known to be selectively inhibited by oxamate [26]. The mitochondria was incubated with lactate in the presence of oxamate. The consumption of lactate was sharply reduced in mitochondria exposed to oxamate (Figure 6). The presence of LDH in the mitochondria was subsequently visualized by BN PAGE and immunoblot analysis. In-gel activity staining revealed two active LDH enzymes within the mitochondria isolated from this astrocytic cell line (Figure 7I). The incubation of gel slabs in NAD^+^ yielded an intense formazan precipitate at the site of immobilized enzyme activity attributed to LDH (Figure 7I, lanes D and E). The specificity of the observed activity bands was confirmed by performing reactions in the absence of NAD^+^ (Figure 7I, lane C). Since two activity bands were generated, AgNO_3 _was utilized to characterize these activity bands. AgNO_3_ is a well characterized inhibitor of all LDH isozymes except LDH1 [32]. The inclusion of AgNO_3_ abolished the appearance of the lower band on the activity gel, thus indicating that the upper band was LDH1 (Figure 7I, lane F). The identification of LDH was further aided by running a porcine heart and muscle standards, which respectively harbours the LDH1 and LDH5 isoforms predominantly (Figure 7I, Lane B) [36], [37]. A similar approach was implemented utilizing the cytosol as a control experiment (Figure 7II). To verify that the observed activity bands were indeed LDH, the bands were precision cut and subjected to two-dimensional immunoblot analysis. Immunoblots were performed with an antibody which does not discriminate between LDH isoforms. Indeed, LDH was detected within the upper and lower activity bands excised from the gel (Figure 8). Thus, two LDH isozymes were detected within the mitochondria isolated from this astrocytic cell line.

**Figure 9:**
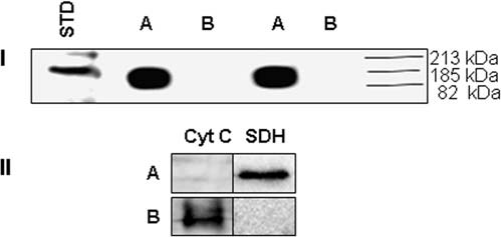
Localization of LDH in the mitochondria. Mitochondria were isolated and separated into A) Mitoplast and B) Outer membrane and inner membrane space fractions. I) Immunoblot for LDH1 in the mitochondrial fractions. II) Immunoblot for Cyt C and SDH to determine purity of mitochondrial fractions. Std corresponds to LDH from porcine heart (Sigma).

LDH1 has been shown to preferentially oxidize lactate to pyruvate [38]. The identification of LDH1 in the mitochondria further confirms that lactate can be utilized as an energy source. Since LDH1 was identified in the mitochondria, it was important to localize this enzyme within the mitochondria. Mitochondria were fractionated using digitonin in order to separate the outermembrane and the intermembrane space fraction from the mitoplast (inner membrane and matrix). These fractions were subsequently probed for LDH1. The purity of each fraction was assessed by immunoblotting for Cyt C (intermembrane space protein) and SDH (mitoplast protein) (Figure 9II). Immunoblot analysis of the two mitochondrial compartments using an anti-LDH1 antibody revealed the occurrence of LDH1 in the mitoplast fraction suggesting that this isoenzyme preferentially localizes to the inner mitochondrial/matrix compartment (Figure 9I).

**Figure 10:**
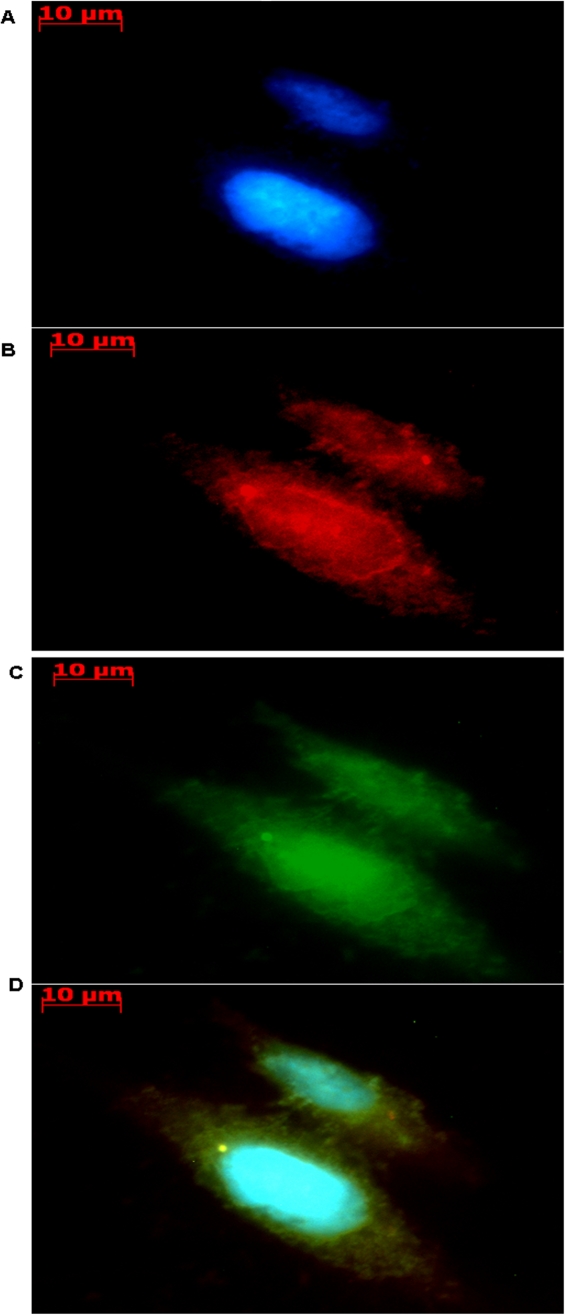
Lactate dehydrogenase localization in an astrocytic cell line. A) Hoechst stain for the nucleus. B) Rhodamine B stain utilized for mitochondrial localization. C) FITC tagged secondary for anti-LDH. D) Merged image of Hoechst, Rhodamine B, and FITC. Note: Yellow spots are indicative of LDH associated with the mitochondria.

The localization of LDH to the mitochondria prompted us to visualize this enzyme using fluorescence microscopy. This astrocytic model cell was probed with polyclonal antibodies conjugated to FITC in order to discern the intracellular localization of this enzyme. Cells were also stained with Rhodamine B, a mitochondrial indicator, in order to correlate the fluorescence of the FITC with the mitochondria. As shown in Figure 10, the green fluorescence attributed to LDH matched the red fluorescence of the mitochondria. Superimposition of the images provided a clearer picture. A strong yellow fluorescence as a consequence of merging the green and the red wavelengths allowed the visualization of the putative mitochondrial LDH (Figure 10D). Hence, this evidence provided vivid proof for the presence of LDH in the mitochondria of this astrocytic cell line. Similar observations in a muscle cell line have been reported [8].

## Discussion

The evidence in this report clearly argues for an important role of lactate in aerobic energy production in the astrocytic cell line (CCF-STTG1). Although further studies with primary cell lines and invivo model systems may shed more light on this observation, this model study provides a fascinating picture as to how lactate may be metabolized in the presence of O_2_. For a long time this metabolite was considered a by-product of anaerobic glycolysis whose accumulation is linked to a variety of biochemical abnormalities [39], [40]. However, recent work has unravelled the mitochondrial utilization of this monocarboxylic acid in numerous cells [41]. For instance, supplementation of perfused rat heart with labeled lactate has been shown to preferentially generate acetyl CoA within the mitochondria [12], [42]. This is the first demonstration of LDH associated with mitochondrial energy production in an astrocytic cell line. Even though astroglia cells are known to supply this monocarboxylate to neurons for its conversion into ATP aerobically, its mitochondrial utilization in brain cells has not been conclusively demonstrated [7], [12], [16]. During sustained periods of neuronal activity, lactate increases several fold in the human brain and remains elevated following stimulation [43]. The astrocytes outnumber neurons 10:1 and the presence of LDH in the mitochondria will allow both the rapid clearance of this metabolite and its use in energy production. Indeed in this study, lactate was readily consumed by this model astrocytic cell with the concomitant production of TCA cycle metabolites, NADH and ATP in an O_2_-dependent manner. Furthermore, the utilization of this monocarboxylic acid was severely impeded by oxamate, a compound known to inhibit LDH. The ^13^C-labelling pattern of the various metabolites would indicate the metabolism of lactate in the mitochondria via the TCA cycle. The ability of lactate-exposed mitochondria to consume O_2_ and to produce ATP would strongly suggest the involvement of this metabolite in oxidative phosphorylation.

Astrocytes require a readily oxidizable source of energy as they actively expend ATP to support a variety of neuronal functions. In the peripheral processes which surround the synapse, D-glucose and glycogen are metabolized anaerobically to lactate in order to generate the ATP required to restore ionic gradients [44]–[46]. However, in the cell body and the larger astrocytic processes which surround the capillaries, the lactate may be utilized as an energy source in the mitochondria [4]. The mitochondria-enriched portions of the astrocytes are in close contact with lactate in the blood stream and in the brain and can thus access this available source of aerobic energy. Indeed, the ability of cells and tissues to use lactate as an aerobic energy source has been correlated to mitochondrial density [4]. Hence, the presence of LDH in the mitochondria of astrocytes would fulfill the dual roles of generating ATP and maintaining lactate homeostasis following neuronal stimulation. Furthermore, it is more accessible to the mitochondria for metabolism than D-glucose. The latter has to be readily metabolized through glycolysis, a process that necessitates the participation of numerous enzymes prior to its utilization by the mitochondria. The evidence generated in this astrocytic cell line clearly illustrates the importance of lactate aerobic metabolism.

In this study, experiments with AgNO_3_, an inhibitor of all isoforms of LDH except LDH1, and monoclonal antibodies specific for LDH1 revealed that this isozyme was an important constituent of this astrocytic mitochondria. This is very interesting as LDH1 is known to preferentially catalyze the conversion of lactate into pyruvate [38]. This intracellular lactate shuttle would allow for the production of one extra NADH, a potential source of 3ATP. Furthermore, this intracellular movement of lactate would restore cytosolic redox, allowing glycolysis to proceed, and would provide a substrate for mitochondrial respiration. The glycerol-3-phosphate and the malate/aspartate shuttles involved in the trafficking of cytoplasmic NADH into the mitochondria have been poorly characterized in astrocytes [47]–[49]. Thus, it is tempting to postulate that the presence of LDH in the mitochondria will effectively help transport NADH with the concomitant release of pyruvate that can be metabolized by the TCA cycle. Furthermore, this pyruvate may potentially act as an antioxidant that can relieve the brain from the constant threat of oxidative stress [50], [51]. Hence, lactate may contribute to the redox potential of the mitochondria. We have recently shown an intriguing role for the TCA cycle and α-ketoglutarate in the homeostasis of ROS [52]. Hence, the presence of a mitochondrial LDH may have the dual role of enhancing ATP production and modulating cellular redox potential.

The fluorescence microscopic studies in this work further revealed the localization of LDH within the mitochondria. In muscle cells, it has been demonstrated that lactate is transported by monocarboxylate transporter (MCT1) into the mitochondrial matrix. In this instance the close association between LDH, localized in the inner membrane space/outermembrane region and the transporter appears to render this process efficient [8]. It is suggested that this proximity enables the rapid oxidation of lactate. In the present study, mitochondrial fractionation and western blot analysis revealed that LDH1 isozyme populated predominantly the matrix/innermembrane components. Although the exact location of the LDH isozymes have not been fully established, it is quite possible that the two LDH isozymes may preferentially localize to different mitochondrial compartments in order to modulated lactate and pyruvate homeostasis on either side of the inner mitochondrial membrane. This disparate spatial localization of LDH may help this organelle metabolize lactate effectively. Besides being involved in energy metabolism, this enzyme appears to participate in a plethora of cellular processes such as β-oxidation and DNA repair [9], [53]. It may be within the realm of possibilities that the presence of this enzyme may have a protective role in the mitochondrion, an organelle known to harbour nuclear material. Hence, the intimate association of LDH with these astrocytic mitochondria may fulfill the enormous energy and oxidative burden inherent in the proper functioning of the brain.

**Figure 11:**
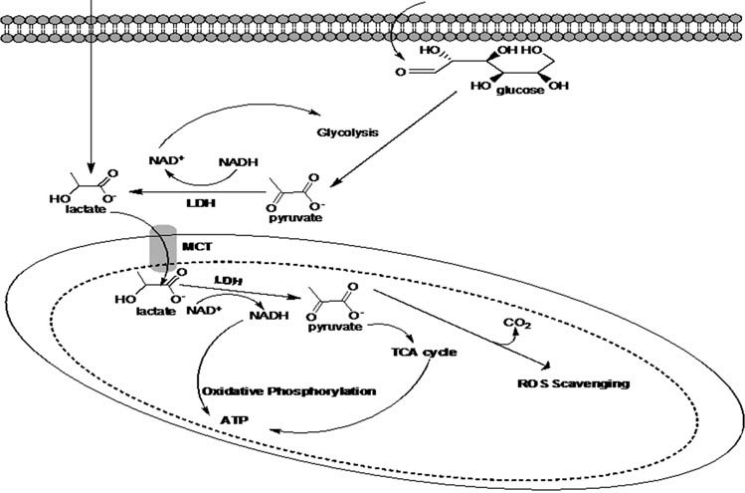
The versatile role of a mitochondrial LDH in an astrocytic cell line. Lactate may be obtained either directly via uptake or by conversion of glucose through the glycolytic pathways.

This first demonstration of LDH in the mitochondria of an astrocytic cell line may indeed help explain the importance of lactate in brain biochemistry. The aerobic metabolism of this monocarboxylic acid will allow efficient generation of ATP, maintain lactate homeostasis, and modulate redox potential. Furthermore, LDH may have a protective role by contributing to DNA repair processes. Hence, this intimate relationship between lactate and the mitochondria will impart multiple benefits to a high-energy demanding organ like the brain. Figure 11 illustrates the importance of mitochondrial lactate metabolism in this astrocytic cell line.
